# Supplementary material for: Predicting visual function by interpreting a neuronal wiring diagram
Source: Nature. 2024 Oct 2;634(8032):113–23. doi: 10.1038/s41586-024-07953-5 (PMC11446822; doi:10.1038/s41586-024-07953-5)
Supplement: Supplementary file 6 — CRF and ERF predictions for individual TmY4 and TmY9 cells. Analogous to Supplementary Data 3, but for TmY target types. Shown are the top four monosynaptic pathways, the strongest pathway passing through each of the top ten intermediary types (ranking from Extended Data Fig. 7), and the trisynaptic pathway Tm1–TmY–Dm3–TmY (see the section entitled Prediction of spatial normalization). [file 41586_2024_7953_MOESM6_ESM.zip › DataS4/TmY9q/720575940618233627.pdf]

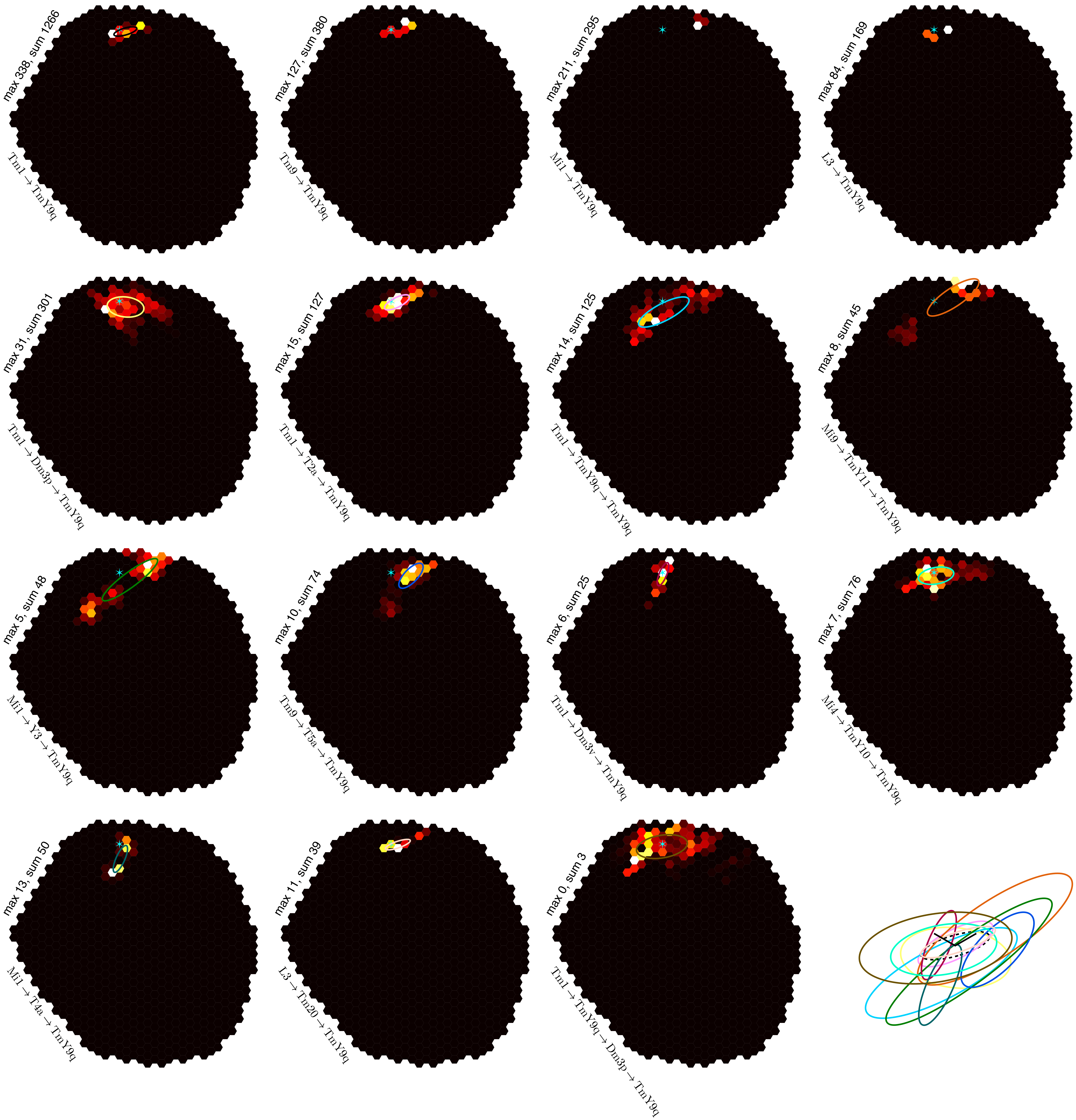

max 338, sum 1266
Tm1 → TmY9q
max 127, sum 380
Tm9 → TmY9q
max 211, sum 295
Mi1 → TmY9q
max 84, sum 169
L3 → TmY9q
max 31, sum 301
Tm1 → Dm3p → TmY9q
max 15, sum 127
Tm1 → T2a → TmY9q
max 14, sum 125
Tm1 → TmY9q → TmY9q
max 8, sum 45
Mi9 → TmY11 → TmY9q
max 5, sum 48
Mi1 → Y3 → TmY9q
max 10, sum 74
Tm9 → T5a → TmY9q
max 6, sum 25
Tm1 → Dm3v → TmY9q
max 7, sum 76
Mi4 → TmY10 → TmY9q
max 13, sum 50
Mi1 → T4a → TmY9q
max 11, sum 39
L3 → Tm20 → TmY9q
max 0, sum 3
Tm1 → TmY9q → Dm3p → TmY9q
